# The effect of GPi-DBS assessed by gait analysis in DYT11 dystonia: a case study

**DOI:** 10.1007/s10072-023-07063-6

**Published:** 2023-09-13

**Authors:** Francesca Lunardini, Sara Satolli, Vincenzo Levi, Davide Rossi Sebastiano, Giovanna Simonetta Zorzi

**Affiliations:** 1 Department of Child Neurology, Child Neuropsychiatry Unit, Fondazione IRCCS Istituto Neurologico Carlo Besta, Via Giovanni Celoria 11, 20133 Milan, Italy; 2 Department of Neurosurgery, Functional Neurosurgery Unit, Fondazione IRCCS Istituto Neurologico Carlo Besta, Milan, Italy; 3 Neurophysiology Unit, Fondazione IRCCS Istituto Neurologico Carlo Besta, Milan, Italy

Dear editor in chief,

Myoclonus-dystonia is characterized by early onset myoclonus and dystonia with genetic heterogeneity; the main causative gene—Ɛ-sarcoglycan (SGCE)—accounts for 70–80% of cases [1].

A recent systematic review [2] supports the strong potential benefits of deep brain stimulation (DBS) of the globus pallidus internus (GPi) in children with monogenic non-degenerative dystonias, with subjects affected by TOR1A (*DYT1*) and SCGE (*DYT11*) dystonias experiencing the most favorable outcomes. However, DBS treatment guidelines for such population are not well established. Among the open challenges, there is the need for quantitative and objective assessment [3] able to juxtapose clinical scoring.

In this work, the positive outcome of a child with *DYT11* dystonia to GPi stimulation is documented through clinical assessment and instrumented gait analysis.

## Methods

### Case history

The child was recruited from the Department of Pediatric Neuroscience of Fondazione IRCCS Istituto Neurologico C. Besta (Milan, Italy). Familial history was negative. His symptoms started at the age of 3, with upper limb involuntary jerky movements and abnormal postures during writing and eating. Genetic tests identified heterozygous pathogenic variant in the intron/exon junction 2 (c.233–1 G > T) of the SGCE gene, inherited from the asymptomatic father. Over the next years, myoclonus and dystonia gradually worsened to involve the head, cranial muscles, and lower limbs. He was treated with clonazepam (0.3 mg/daily), trihexyphenidyl (6 mg/daily), and zonisamide (150 mg/daily), without benefit. Hence, at the age of 13, GPi-DBS was proposed.

Under general anesthesia, the patient underwent bilateral implantation of a quadripolar electrode (3387 Medtronic) in the GPi and of a rechargeable pulse generator (Activa RC, Medtronic) in the right subclavian region. The Leksell Vantage Stereotactic System (Elekta), five bilateral microelectrode recordings, and intra-operative imaging acquisition via the O-arm portable CT scan (Medtronic) were used to place and confirm lead correct placement. GPi coordinates related to the anterior commissure–posterior commissure (AC-PC) midpoint were set at X (± 19), Y (+ 2), and Z (− 4). After 3 days, stimulation was started using the two central monopolar contacts for each side (parameters: voltage: 1 V; frequency: 130 Hz; pulse width: 60 µs). At follow-up, voltage was increased up to 2.2 V with significant benefit and without side effects.

Before surgery and at 6-month follow-up, the patient was videotape assessed by two raters (GZ and SS) with the Burke-Fahn-Marsden Dystonia Rating Scale (BFMDRS) and the Unified Myoclonus Rating Scale (UMRS).

### Experimental procedure

The patient underwent two sessions of gait analysis: 3 days before surgery and 6 months after surgery (with stimulator on). Each session included at least 5 trials, where the patient walked along a 7-m walkway at his preferred speed. At least 10 gait cycles for each limb were selected based on the quality of the marker trajectories.

The walking was analyzed with the BTS Bioengineering system, which synchronizes 8 infrared cameras (SMART-D; sampling frequency (fs) = 70 Hz), and 8-channel electromyography (EMG) (Free1000; fs = 1000 Hz). To study joint kinematics and spatiotemporal gait parameters, 22 passive markers were positioned on the subject according to the Davis protocol [4]. To study muscle activations, surface electrodes were placed, in bipolar configuration (interelectrode distance: 10 mm), on 8 lower-limb muscles: rectus femoris (RF), semitendinosus (ST), tibialis anterior (TA), and soleus (SO) on both limbs.

Fondazione IRCCS Istituto Besta Ethical Committee approved the study protocol (n. 78/2020). Parents gave informed written consent, and the patient gave written assent. The research was performed in accordance with the Declaration of Helsinki.

### Data analysis

Data analysis was conducted with Smart Analyzer (BTS Bioengineering) and MATLAB® R2021a (MathWorks®) software. We extracted the following spatiotemporal gait parameters for each limb and session: *Stride Length*, *Step Length*, *Stride Time*, *Stance Time* and *Percentage*, *Swing Time* and *Percentage*, and *Speed*. *Cadence* was estimated for each session.

Joint angles (the hip, knee, and ankle in sagittal plane; the ankle in transverse plane) during the gait cycle were compared with normative curves with Pearson’s correlation coefficient, for both limbs and sessions.

As for EMG, after applying a band-pass Butterworth filter (5th order, 20–450 Hz), signal envelopes were extracted with halfway rectification followed by low-pass Butterworth filtering (5th order, 5 Hz). Each channel was normalized by the maximum activation during the entire task. For each muscle, we averaged the envelope over all gait cycles and compared it with normative muscle activation gait patterns [5].

**Fig. 1:**
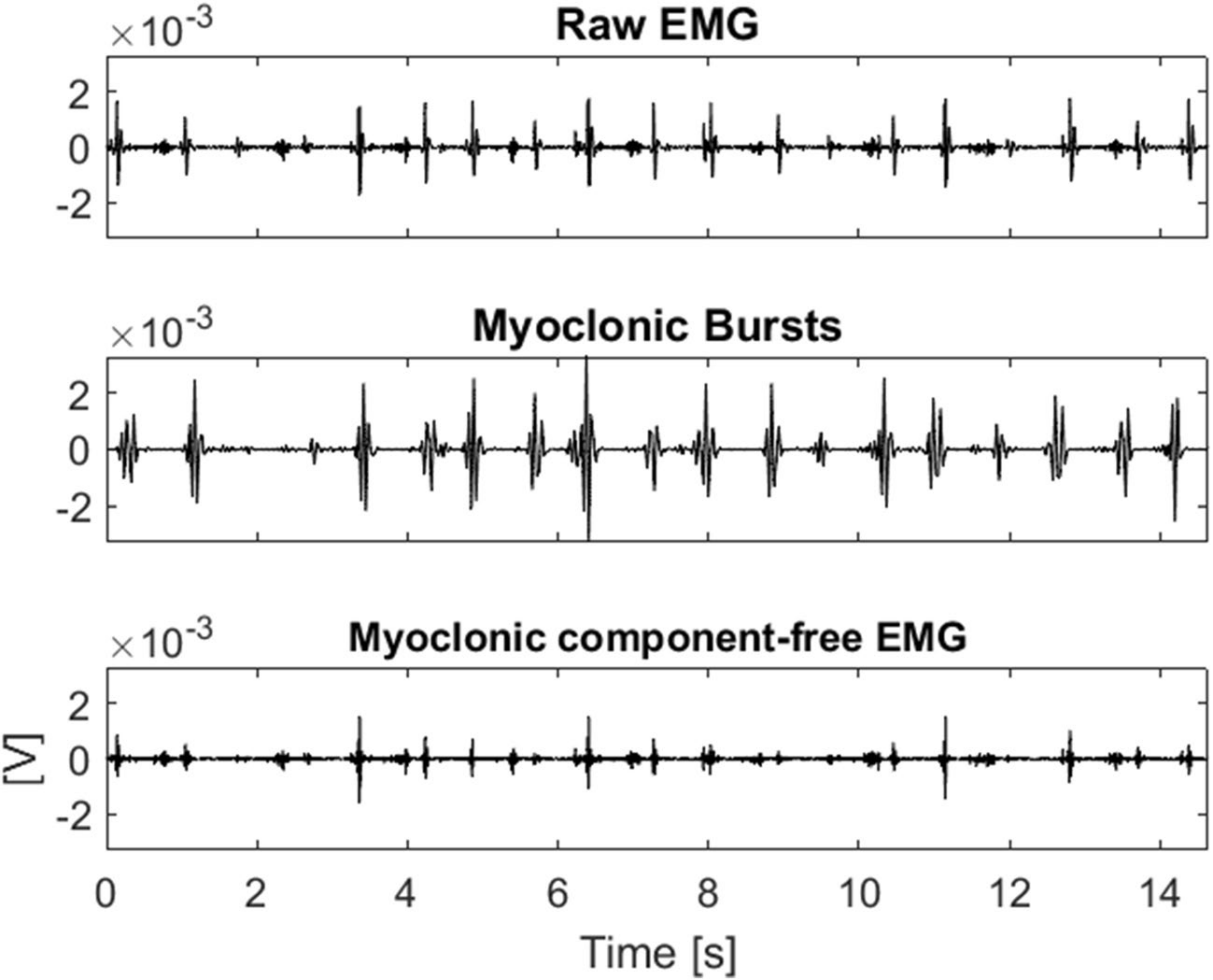
Myoclonic burst separation for the left soleus (SO) muscle: the first row presents the raw EMG signal; the second row shows the myoclonic component obtained with wavelet decomposition; the third row presents the EMG signal free from myoclonic bursts

We then exploited wavelet decomposition to identify and separate the myoclonic component from the EMG signal. For each muscle, we performed a 5-level wavelet decomposition of the raw EMG signal using the 8th-order Daubechies wavelet. The obtained approximation signal is able to capture and separate the myoclonic bursts (Fig. 1). After that, the envelope of the myoclonic component was extracted with the same filtering used for the original EMG signal.

## Results

### Clinical evaluation

Clinical scores demonstrate a great improvement in both dystonia and myoclonus (Table 1).

**Table 1:** Clinical scores at baseline and at 6-month follow-up

Burke-Fahn-Marsden Dystonia Rating Scale				
	Baseline		6-month follow-up	
	Severity	Provoking	Severity	Provoking
Eyes	0	0	0	0
Mouth	0	0	0	0
Speech	0	0	0	0
Neck	2	2	1	1
Right arm	0	0	1	1
Left arm	0	0	0	0
Trunk	1	1	0	0
Right leg	1	2	1	1
Left leg	2	1	0	0
	TOT = 10		TOT = 3	
The Unified Myoclonus Rating Scale section				
	Baseline		6-month follow-up	
	At rest			
	Frequency	Amplitude	Frequency	Amplitude
Upper face	0	0	0	0
Lower face	3	2	1	1
Neck	3	2	1	1
Trunk	0	0	0	0
Right arm	3	2	2	2
Left arm	3	2	2	2
Right leg	2	1	0	0
Left leg	2	1	0	0
	TOT = 28		TOT = 10	
The Unified Myoclonus Rating Scale section				
	Baseline		6-month follow-up	
	With action			
	Frequency	Amplitude	Frequency	Amplitude
Eyelids	4	2	1	1
Neck	4	1	0	0
Trunk	2	1	1	1
Right arm	4	2	4	1
Left arm	4	2	4	1
Right leg	1	1	0	0
Left leg	1	1	0	0
Arising	0	0	0	0
Standing	4	2	2	1
Walking	0	0	0	0
	TOT = 40		TOT = 12	

At baseline, the patient had myoclonic jerks involving the face, head, and upper limbs, present at rest and exacerbated by action. There was also neck and trunk dystonia and action-induced dystonia of the right upper limb (during writing and eating). Gait was severely affected, and running was impossible, due to marked action dystonia with superimposed myoclonic jerk of lower limbs, more pronounced on the right side.

At follow-up, the patient showed lower frequency of jerks, mostly located in upper limbs during task execution and while maintaining postures. Fewer jerks were also present in the facial muscles, head, and trunk. The patient no longer showed lower limb dystonia during gait, but only milder dystonia in the right upper and lower limbs while running. A slight cervical dystonia persisted.

The video documenting the clinical status of the patient at baseline and at follow-up is presented as Supplementary Material (Video 1).

### Instrumented gait analysis

The video documenting the patient’s gait analysis at baseline and at follow-up is presented as Supplementary Material (Video 2).

#### Spatiotemporal gait parameters

At baseline, we observe an altered gait, with decreased *Step Length*, increased timing, and low *Speed*. All parameters strongly improve at follow-up, except for the *Percentage of Stance* and *Swing* phases, which presented a correct ratio also at baseline. In both sessions, gait was quite symmetrical (Table 2).

**Table 2:** Spatiotemporal parameters of gait at baseline and at 6-month follow-up (median values and interquartile ranges). Reference normative values are reported

Variable	Baseline		Normative reference	6-month follow-up	
Cadence (steps/min)	76.2 (1.95)		129.6	113.4 (2.7)	
Stride Length (m)	1 (0.09)	1.02 (0.12)	1.13	1.38 (0.03)	1.39 (0.04)
Step Length (m)	0.55 (0.05)	0.48 (0.05)	0.58	0.71 (0.03)	0.68 (0.02)
Stride Time (s)	1.58 (0.11)	1.54 (0.11)	0.93	1.04 (0.06)	1.06 (0.04)
Stance Time (s)	0.92 (0.06)	0.9 (0.02)	0.54	0.62 (0.03)	0.61 (0.02)
Swing Time (s)	0.63 (0.05)	0.64 (0.09)	0.39	0.44 (0.03)	0.43 (0.03)
Stance (%)	58.46 (1.46)	56.91 (2.61)	57.97	58.5 (1.37)	59.01 (1.02)
Swing (%)	40.79 (1.19)	41.69 (2.45)	42.03	41.61 (1.69)	40.99 (0.47)
Velocity (m/s)	0.6 (0.1)	0.7 (0.1)	1.2	1.4 (0.1)	1.3 (0.1)

#### Kinematics and EMG

**Fig. 2:**
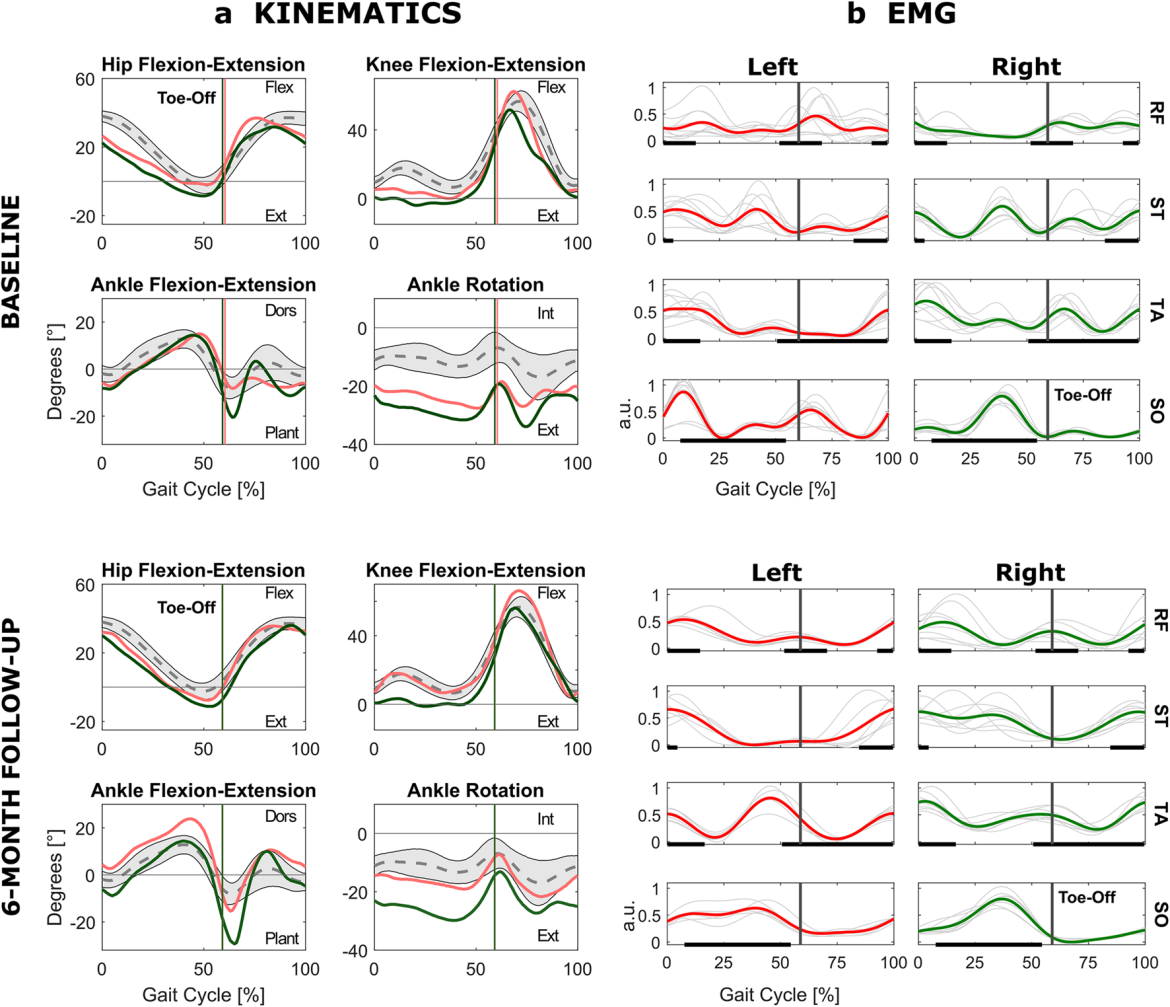
**a** Kinematics. Lower limb joint angles (hip flexion–extension, knee flexion–extension, ankle flexion–extension and rotation) over the gait cycle at baseline (upper panel) and at 6-month follow-up (lower panel). Red and green curves represent the average angles for the left and right side, respectively. Normative ranges are presented in grey. **b** EMG. EMG patterns (rectus femoris (RF), semitendinosus (ST), tibialis anterior (TA), and soleus (SO)) over the gait cycle at baseline (upper panel) and at 6-month follow-up (lower panel). For each limb, tick curves represent the mean muscle envelopes (averaged over the single gait cycles (thin gray)) for each limb. The tick black horizontal lines on the *x*-axis indicate the portion of the gait cycle in which the muscle is supposed to be active, according to normative references

**Fig. 3:**
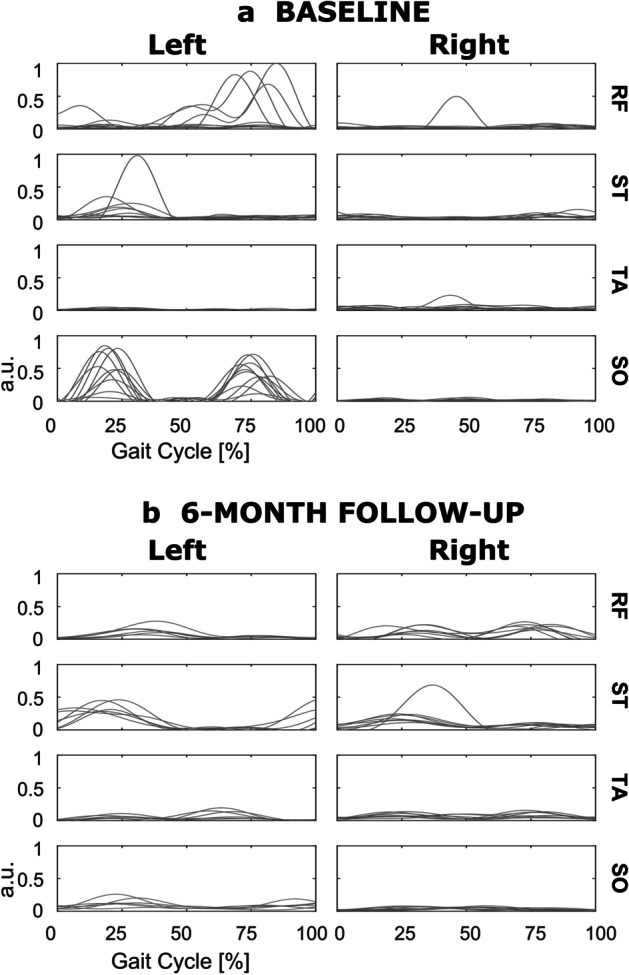
EMG myoclonic component (left and right rectus femoris (RF), semitendinosus (ST), tibialis anterior (TA), and soleus (SO)) over the gait cycle at baseline (**a**) and at 6-month follow-up (**b**). For each muscle, the envelope of myoclonic component over all gait cycles is presented

We compared lower limb joint angles (Fig. 2), EMG patterns (Fig. 2), and myoclonic activity (Fig. 3) during gait at baseline and at follow-up.

At baseline, joint angle alterations are similar for both limbs. In early stance, the patient presents reduced hip and knee flexion, coherently with an atypical activation pattern of RF and ST muscles, resulting in reduced *Step Length*. Both limbs show an overall excessive ankle lateral rotation and an irregular plantar/dorsiflexion during swing. On the left side, the marked plantarflexion is explained by an altered pattern of SO and TA muscles. Myoclonic bursts are strongly present in the left limb muscles, especially in the SO.

At follow-up, in line with the improvement of the spatiotemporal parameters, we notice more regular EMG activations, with reduced between-cycle variability. Joint angle patterns improve, as confirmed by higher values in the correlation coefficients between the patient’s joint angles and the normative curves (Table 3). A more marked change is visible in the left side, with a correct activation of the RF-ST muscles, resulting in hip and knee flexion–extension angles in line with the normative reference. The ankle joint improves, with a reduced lateral rotation and a more correct plantar/dorsiflexion angle during swing, although the TA activation during stance causes an excessive dorsiflexion. Myoclonic activity is reduced, but some bursts persist especially for left ST. On the right side, ST activation still shows some irregularities and, although an improvement in the hip flexion/extension pattern is present, a lacking knee flexion after weight acceptance persists. A marked ankle lateral rotation persists, but plantar/dorsiflexion during swing improves.

**Table 3:** Correlation between the patient’s joint angle traces and the normative reference curves

	Correlation (Rho)			
	Baseline		6-month follow-up	
Joint angle	Left	Right	Left	Right
Hip flexion/extension	0.83	0.87	0.97	0.98
Knee flexion/extension	0.97	0.95	0.99	0.97
Ankle flexion/extension	0.79	0.90	0.95	0.90
Ankle internal/external rotation	0.30	0.44	0.92	0.61

## Discussion

This work presents the positive outcome of GPi stimulation in a 13-year-old boy with *DYT11* dystonia.

The patient, after 6 months, showed great improvement of symptoms, documented by clinical scores.

Instrumented gait analysis was conducted to investigate how DBS improves walking through objective information about spatiotemporal parameters and joint angle motion. For an overarching understanding of the reasons behind gait abnormalities, surface EMG was leveraged to study the timing and action of muscles and myoclonic activity. The instrumented assessment revealed a strong significant improvement of the patient’s gait: at follow-up, the spatiotemporal parameters for both limbs present values in line with the normative reference, outlining a performance characterized by increased cadence and step length, which contribute to an enhanced walking speed. Data suggest that the reason underlying this improvement can be found in less variable and more regular muscle activation patterns and reduced myoclonic activity, which in turn contribute to joint angle motions in line with normative references.

While both clinical and instrumented assessment are able to capture the improvement following GPi stimulation, instrumented analysis only reveals that, at baseline, irregular muscle activation patterns and myoclonic activity most severely affect the left limb and, although this limb shows the most marked improvement, irregularities persist after DBS. Additional follow-up visits are necessary to assess long-term outcomes [6].

To our knowledge, this is the first report that evaluates GPi-DBS effect on a boy with *DYT11* dystonia through both clinical scores and instrumented assessment. Our results suggest that gait analysis comprising spatiotemporal, kinematic, and EMG measures can be a promising and noninvasive tool to gain insights into DBS improvements that are only partially captured by clinical scores.

Quantitative objective assessment tools can be leveraged to optimize DBS intervention, for instance, to guide the choice of the stimulation parameters. Further studies in a larger pediatric dystonic population with diverse etiologies—to enhance the quantification and characterization of DBS treatment response—can support in the creation of a consensus regarding when, if at all, DBS should be performed for patients with various causes of dystonia.

### Supplementary Information

Below is the link to the electronic supplementary material.

Video 1 (MP4 187067 KB)

Video 2 (MP4 6119 KB)
